# *Photorhabdus luminescens *genes induced upon insect infection

**DOI:** 10.1186/1471-2164-9-229

**Published:** 2008-05-19

**Authors:** Anna Münch, Lavinia Stingl, Kirsten Jung, Ralf Heermann

**Affiliations:** 1 Ludwig-Maximilians-Universität München, Department Biologie I, Bereich Mikrobiologie, Maria-Ward-Str. 1a, D-80638 München, Germany; 2 Munich Center for Integrated Protein Science (CIPSM), Ludwig-Maximilians-Universität München, München, Germany; 3 Ludwig-Maximilians-Universität München, Adolf-Butenandt-Institut, Bereich Biochemie, Schillerstr. 44, 80336 München, Germany; 4 Universitätsklinikum Würzburg, Klinik und Poliklinik für Strahlentherapie, Josef-Schneider-Str. 11, 97080 Würzburg, Germany

## Abstract

**Background:**

*Photorhabdus luminescens *is a Gram-negative luminescent enterobacterium and a symbiote to soil nematodes belonging to the species *Heterorhabditis bacteriophora*. *P*.*luminescens *is simultaneously highly pathogenic to insects. This bacterium exhibits a complex life cycle, including one symbiotic stage characterized by colonization of the upper nematode gut, and a pathogenic stage, characterized by release from the nematode into the hemocoel of insect larvae, resulting in rapid insect death caused by bacterial toxins. *P. luminescens *appears to sense and adapt to the novel host environment upon changing hosts, which facilitates the production of factors involved in survival within the host, host-killing, and -exploitation.

**Results:**

A differential fluorescence induction (DFI) approach was applied to identify genes that are up-regulated in the bacterium after infection of the insect host *Galleria mellonella*. For this purpose, a *P. luminescens *promoter-trap library utilizing the mCherry fluorophore as a reporter was constructed, and approximately 13,000 clones were screened for fluorescence induction in the presence of a *G. mellonella *larvae homogenate. Since *P. luminescens *has a variety of regulators that potentially sense chemical molecules, like hormones, the screen for up-regulated genes or operons was performed *in vitro*, excluding physicochemical signals like oxygen, temperature or osmolarity as variables. Clones (18) were obtained exhibiting at least 2.5-fold induced fluorescence and regarded as specific responders to insect homogenate. In combination with a bioinformatics approach, sequence motifs were identified in these DNA-fragments that are similar to 29 different promoters within the *P. luminescens *genome. By cloning each of the predicted promoters upstream of the reporter gene, induction was verified for 27 promoters *in vitro*, and for 24 promoters in viable *G. mellonella *larvae. Among the validated promoters are some known to regulate the expression of toxin genes, including *tccC1 *(encoding an insecticidal toxin complex), and others encoding putative toxins. A comparably high number of metabolic genes or operons were observed to be induced upon infection; among these were *eutABC*, *hutUH*, and *agaZSVCD*, which encode proteins involved in ethanolamine, histidine and tagatose degradation, respectively. The results reflect rearrangements in metabolism and the use of other metabolites available from the insect. Furthermore, enhanced activity of promoters controlling the expression of genes encoding enzymes linked to antibiotic production and/or resistance was observed. Antibiotic production and resistance may influence competition with other bacteria, and thus might be important for a successful infection. Lastly, several genes of unknown function were identified that may represent novel pathogenicity factors.

**Conclusion:**

We show that a DFI screen is useful for identifying genes or operons induced by chemical stimuli, such as diluted insect homogenate. A bioinformatics comparison of motifs similar to known promoters is a powerful tool for identifying regulated genes or operons. We conclude that signals for the regulation of those genes or operons induced in *P. luminescens *upon insect infection may represent a wide variety of compounds that make up the insect host. Our results provide insight into the complex response to the host that occurs in a bacterial pathogen, particularly reflecting the potential for metabolic shifts and other specific changes associated with virulence.

## Background

*Photorhabdus luminescens *is an entomopathogenic enterobacterium that exists in a state of mutualistic symbiosis with nematodes of the family Heterorhabditidae. This bacterium is found in the gut of free-living, infective *Heterorhabditis bacteriophora *juveniles. Upon entering an insect host, the nematodes release the bacteria by regurgitation directly into the insect's hemocoel. Once inside the hemocoel, the bacteria replicate rapidly and cause lethal sepsis in the host by producing different toxins that kill the insect within 48–72 hours. Bioconversion of the insect's body by *P. luminescens *produces a rich food source for the bacteria, and for the nematode. Nematode reproduction is supported by *P. luminescens*, probably because essential nutrients that are required for efficient nematode proliferation within the insect host are produced by the bacteria [1]. Furthermore, *P. luminescens *produces antibiotics that may protect the insect cadaver from infection by other bacteria. When the insect cadaver is depleted, the nematode/*P. luminescens *symbiotes leave the carcass in search for a new insect host [2,3].

*Photorhabdus *species exist in two forms, primary and secondary variants, which differ in morphological and physiological traits. Primary variants produce extracellular protease, extracellular lipase, intracellular protein crystals CipA and CipB, antibiotics, and are bioluminescent. Secondary variants lack protease, lipase, antibiotic activity, and bioluminescence is strongly decreased; they differ further in colony morphology, pigmentation, dye adsorption, metabolism, and the ability to support nematode growth and reproduction. It is hypothesized that primary variants correspond to the nematode-associated and insect-infective form, and secondary variants correspond to late stationary phase cells in infected insects and the re-associative form of the bacteria [4-6]. The genome of *P. luminescens *strain TT01 has been fully sequenced; it contains more potential virulence genes than any other bacterial genome sequenced to date [7].

When *P. luminescens *is released from the nematode into the insect's hemolymph, the environmental conditions for the bacteria change dramatically. For an effective infection, *P. luminescens *must sense the conditions in the new host and to switch to the pathogenic lifestyle. Pathogenesis is characterized by evasion of the insect's immune response and host death by toxin production. Insect cellular immunity includes circulating hemocytes that recognize foreign material and facilitate encapsulation of invading microbes. The resultant capsule is melanized through the action of the enzyme phenoloxidase, and is then removed from the hemolymph [8,9]. Furthermore, insects produce a range of antimicrobial peptides that can directly target bacterial membranes [10]. *P. luminescens *is not believed to be inherently resistant to the insect's immune response [11]. Moreover, it is assumed that *P. luminescens *triggers an immune response, but that this response is circumvented and controlled [12]. This assertion is supported by the fact that this bacterial species secretes a metalloprotease, PrtS, which specifically induces melanization of the hemolymph [13]. A small molecule antibiotic produced by *P. luminescens *inhibits phenoloxidase and controls melanization [14]. Another potential immune response circumvention mechanism is via the production of furanosyl-borate diester, autoinducer 2 (AI-2), a signaling molecule that is required for resistance to reactive oxygen species, another component of the insect's early immune response [15].

*P. luminescens *also produces insecticidal compounds, including the insecticidal toxin complex proteins (Tc), which exhibit oral toxicity [16]. Three Tc components are required for full toxicity: a TcdA-like, a TcdB-like, and a TccC-like component [17]. However, neither an enzymatic activity nor detailed mechanism of action for these toxins has been reported. Another virulence factor was identified based on its ability to confer insect pathogenicity to *E. coli *injected into caterpillars. *E. coli *producing 'makes caterpillars floppy' toxin 1, or Mcf1 promote the rapid destruction of the insect midgut, resulting in 'floppy' caterpillars that suffer from a loss of body turgor [18]. Mcf1 mimics BH3-domain-only mitochondrial proapoptotic proteins, and promotes apoptosis in the insect gut and in mammalian cells [19]. Another similar acting toxin, Mcf2, has a domain homologous to the HrmA type-III secretion factor of the plant pathogen *Pseudomonas syringae*; HrmA is known to induce cell death in tobacco [20]. As is the case for *mcf1*, heterologous expression of *mcf2 *in *E. coli *is also sufficient to kill caterpillars [21]. Most recently, the "*Photorhabdus *insect-related" toxins (PirAB) were shown to be binary toxins with injectable and oral activity towards different insects [22]. Although PirB exhibits similarities to Juvenile Hormone Esterase (JHE) of the beetle, *Leptinotarsa decemlineata*, JHE activity was not observed for PirAB, which suggests that the mode of action is different [22].

After insect host death, the cadaver is available as a nutrient source, and successful use of the dead tissue reflects the ability to utilize a broad and complex range of nutrients by the microbe. Generally, little attention has been given to the changes in response to metabolic requirements and substrate availability that occur in *P. luminescens in vivo*. Iron scavenging is important for *P. temperata *strain K122 growth within the insect, which indicates importance for pathogenicity.

When the *exbD *gene encoding the TonB protein involved in iron scavenging was deleted, bacterial growth and virulence were attenuated, but this effect could be reversed by co-injection of iron [23]. Other components present in the insect host hemolymph, including amino acids and phosphatidylethanolamine, are potential nutrients for *P. luminescens*, but evidence for their use is lacking [24].

Little is known about putative signals sensed by the bacteria to identify the host. Two global regulators, HexA and Ner, appear to control the switch between mutualism and pathogenicity in *P. luminescens *[25,26]. Furthermore, the two-component systems PhoQ/PhoP and AstS/AstR have been shown to be involved in the regulation of mutualism and/or pathogenicity gene expression [27,28], and are believed to be members of a putative complex regulation network that coordinates the switch between mutualism and pathogenicity [2]. The signals that stimulate these sensor kinases remain unclear. Generally, the expression of pathogenicity coupled genes is often dependent on physicochemical parameters, including: temperature, oxygen, osmolarity and Mg^2+ ^concentration [29-32]. Recent studies show that microorganisms and their hosts communicate with each other using hormonal signals. This cross-kingdom cell-to-cell signaling involves small molecules that are produced by eukaryotes and hormone-like chemicals that are produced by bacteria, including quorum-sensing autoinducers [33]. *P. luminescens *has a multiplicity of LuxR-like receptors, which are believed to bind molecules produced by the eukaryotic host(s), including hormones and homoserine lactones [7,24]. Since *P. luminescens *lacks homoserine lactone synthase LuxI, it is assumed that the bacteria respond to homoserine lactones produced by other bacteria [24]. Evidence for cross-kingdom signalling between *P. luminescens *and eukaryotic hosts has not yet been presented.

The fundamental mechanisms that facilitate insect infection, survival and host exploitation by *P. luminescens *remain underexplored. We performed a fluorescence-based promoter-trap library screen of *P. luminescens*, combined with a bioinformatics approach to identify promoter motifs and the corresponding genes or operons that are induced upon infection of the insect host *Galleria mellonella*. We focussed on the identification of promoters with enhanced activity in response to insect based stimuli, while physicochemical parameters like pH, oxygen and osmolarity were constant. Using this method we identified 27 responsive genes or operons encoding a wide variety of proteins with known or potential functions that relate to host adaptation or virulence.

## Results

### Construction of a fluorescence-based *P. luminescens *promoter-trap library

**Figure 1:**
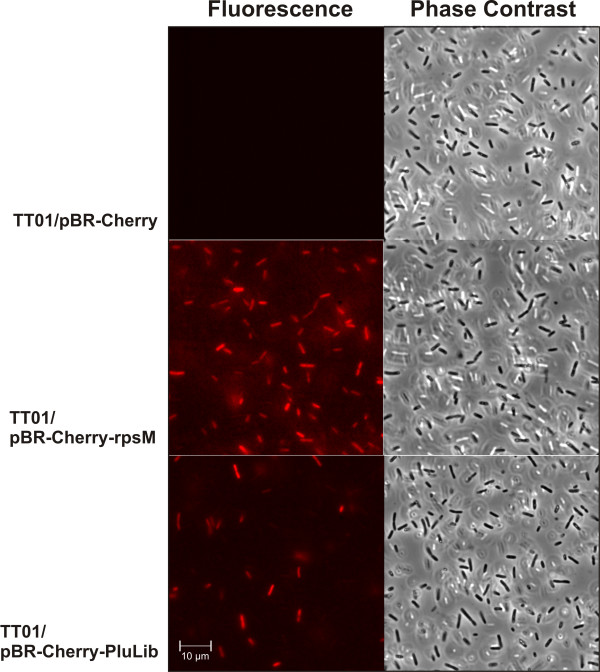
**Cells of *P. luminescens *expressing *mcherry***. *P. luminescens *TT01 transformants carrying plasmid pBR-Cherry (no promoter, negative control), pBR-Cherry-rpsM (*rpsM*-promoter, positive control), and pBR-Cherry-PluLib (promoter-trap library) were cultivated in complex medium. Cells were analyzed by fluorescence- (left panel) and phase contrast microscopy (right panel).

Differential Fluorescence Induction (DFI) of promoter-trap library clones has been proved as a promising method for exploring niche specific bacterial gene expression (see [34] for review). To perform DFI in *P. luminescens*, we cloned the gene encoding Red Fluorescence Protein mCherry, as a reporter gene into plasmid pBR322 (see Methods for detail). As a positive control, the *rpsM *promoter of *P. luminescens *was cloned upstream of the reporter gene. The *rpsM *gene encodes the ribosomal protein S13, and is expressed constitutively in *E. coli *[35]. *P. luminescens *TT01 transformants carrying plasmids pBR-Cherry or pBR-Cherry-rpsM, respectively, were grown in complex medium, and fluorescence was analyzed microscopically. As shown in Fig. 1, only cells expressing from pBR-Cherry-rpsM were fluorescent, in contrast to those harbouring the promoter-less *mcherry *gene (TT01/pBR-Cherry). These data show that this basic tool is useful for the successful construction of a promoter-trap library to perform DFI. The library was constructed that comprises approximately 15,000 clones that harbour the reporter gene downstream of DNA-fragments that range from 300–700 bp in size, and represent 1.5-fold coverage of the *P. luminescens *genome. Among the 15,000 clones was one that formed a visibly red colony due to the presence of the highly active *cipB *promoter controlling the expression of the crystal inclusion protein CipB upstream of *mcherry *(data not shown). The presence of only one such colony in 15,000 reflected a low redundancy in the promoter trap library. For microscopy, cells of the 15,000 clones were mixed, grown in complex medium, and analyzed for fluorescence. As expected, fluorescence intensity for cells harbouring plasmids of the promoter-trap library was more diverse than that of the positive control (*rpsM*-promoter). This is indicative of the fact that different promoters induce or repress reporter expression at varying intensities, resulting in a range of fluorescence intensities across cells (Fig. 1).

### Identification of promoter motifs responsive to insect homogenate

**Figure 2:**
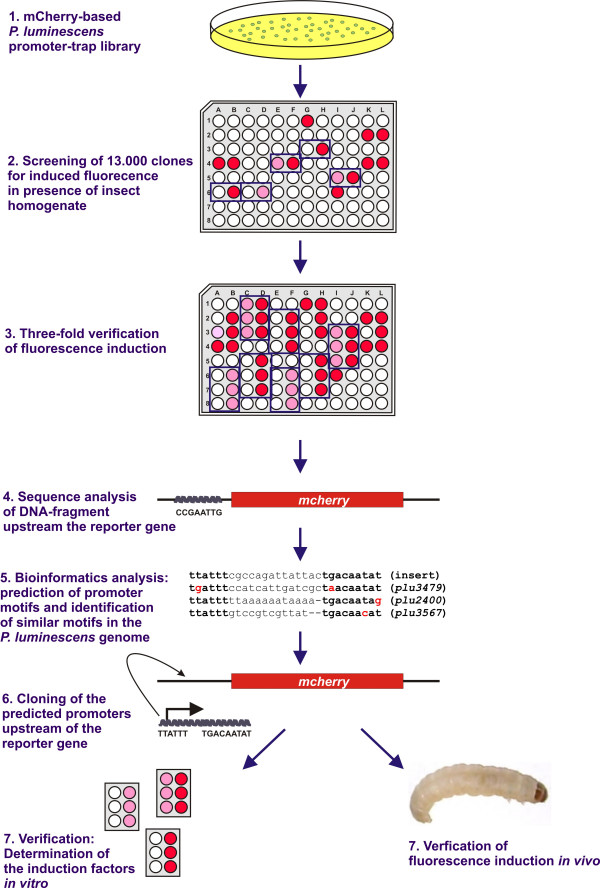
**Schematic presentation of the Differential Fluorescence Induction screen**. Approximately 13,000 clones of the *P. luminescens *promoter-trap library, based on the reporter gene *mcherry *encoding the Red Fluorescent Protein mCherry, were analyzed for induced fluorescence in the presence of *G. mellonella *larvae homogenate. Positive clones were verified in triplicate before sequencing the DNA-fragment upstream of the reporter gene. When no native promoter was present within the DNA-fragment, a promoter motif search was performed. The *P. luminescens *genome was then searched for similar promoter motifs. DNA-sequences containing the predicted promoter motifs were cloned upstream of the reporter gene *mcherry*, and induction was analyzed after exposure of *P. luminescens *to insect homogenate (*in vitro*) or in viable *G. mellonella *larvae (*in vivo*).

DFI with *P. luminescens *cells expressing the promoter-trap library was performed upon exposure of single clones to insect homogenate *in situ *(Fig. 2). Single clones were screened for induced fluorescence in presence of Schneider's insect cell medium containing insect homogenate. DFI was performed in Schneider's medium because it exhibited the lowest background fluorescence compared to other media (data not shown). Verification and sequence identification (see below) were performed in parallel, so that the screen was aborted when the first identified DNA-sequence occurred in duplicate. *P. luminescens *library clones each harbouring one of the promoter-trap library plasmids (pBR-Cherry-PluLib) were inoculated into two wells of a 96-well microtiter plate each, one contained Schneider's medium (non-induced) and one contained Schneider's medium with the *G. mellonella *insect larvae homogenate (induced). Those clones with an at least 2.5 higher fluorescence under induced versus non-induced conditions [36] were collected, and induction was verified by testing each positive clone in triplicate. Only clones that exhibited reliable fluorescence induction in independent experiments (with an induction factor ≥ 2.5) were considered positive, having a promoter upstream of the reporter that was induced by insect-specific signals. Of the 13,000 clones screened, we identified 517 positive clones with 18 of these being validated. For most of the remaining 499 clones, induction factors of ≥ 2.5 were found only once or twice, so these were not considered further (Fig. 2). Compared to other DFI screens where clones with induction factors of 1.5-fold were considered positives [37,38], which may explain the relatively low number of validated positives (18 of 13,000) obtained with a cut-off induction factor of ≥ 2.5, the highest cut-off value observed in the literature [35].

Plasmids from the 18 positive clones were isolated and the sequences upstream of *mcherry *were determined. In four plasmids, the promoters of genes *plu1950*, *plu3608*, *plu3688*, and the *agaZSVCD *operon were identified, whereas in 14 plasmids the sequence of the insert was identified as a protein coding region (Tab. 1). To confirm the host-specific induction of the 18 positive DNA-fragments, we injected each positive clone into the hemocoel of live *G. mellonella *larvae. After 48–72 h the larvae were bled, and the hemolymph was analyzed for fluorescing *P. luminescens *cells. In all 18 cases, the hemolymph contained brightly fluorescent *P. luminescens *cells (data not shown), which indicated that a DNA-motif was present within these DNA-fragments that served as a promoter for the reporter. Using restriction endonuclease DraI for library construction cutting in sequence TTTAAA might possibly result in frequent cutting within intergenic AT-rich promoter sequences, resulting in an enrichment of intragenic sequences. Therefore, we searched for putative promoter motifs (-10 and -35 regions) within these intragenic DNA-fragments by bioinformatics analysis using the software BProm. This software is used for prediction of σ^70 ^promoter motifs with >80% accuracy. We focussed on the identification of promoter motifs belonging to the σ^70 ^family, because this family comprises the majority of *E. coli *promoter motifs, and includes primary and alternative σ-factors. In general, the σ^70 ^promoter motif is more conserved in the -10 and -35 regions, than in the spacer found in between σ^54^-dependent promoters, making a σ^54 ^promoter prediction difficult [39,40].

**Table 1:** Inducible promoter motifs in *P. luminescens *in presence of insect larvae homogenate

**Clone No**.	**Insert/size**	**Fluorescence Induction**	**No. of identical clones**	**Intergenic region**	**Identified promoter motif within the DNA-insert and homology to promoter motif of intergenic regions, [-35 region, -spacer-, -10 region]**
1	*plu1165 *(320 bp)	12.7-fold (± 3.0)	1	No	**ttattt**cgccagattattac**tgacaatat****(insert)**
					**t*g*attt**ccatcattgatcgc**t*a*acaatat ****(*plu3479*)**
					**ttattt**ttaaaaaataaaa-**tgacaata*g *****(*plu2400*)**
					**ttattt**gtccgtcgttat--**tgacaa*c*at**** (*plu3567*)**
					**ttattt**ttggctatttaa--**t*a*acaatat ****(*plu3608*)**

2	*rpoH *(71 bp)	3.2-fold (± 0.3)	1	no	**ttgtta**aagttgcaac**-aactaaag ****(insert)**
					***g*tgtta**gtgttaataa-**aactaaa*a***** (*plu1672*)**
					**t*a*gtta**accacggcgaa**aactaaag**** (*sucA*)**
					***a*tgtta**atttatggtt-**aactaaa*a***** (*plu1864*)**

3	*plu0097 *(385 bp)	2.8-fold (± 0.1)	1	no	**tctatt**ggggacgggcgtcc**-tggtttgat ****(insert)**
					**tc*a*att**tgtcattttggctaa**tggtttgat**** (*hutU*)**

4	*plu1880/plu3515-plu3516 *(243 bp)	3.5-fold (± 0.2)	1	no	**ttaaag**agttgaattcagt**tatttaaat ****(insert)**
					**tt*g*aag**gataacgggta--**tatttaaat**** (*plu4122*)**

5	*plu1375 *(376 bp)	4.3-fold (± 1.2)	1	no	**ttgatg**tggattacgtggtt--**gattatttt**** (insert)**
					**ttgat*t***agcctgaattacaggg**gattatttt**** (*plu1463*)**

6	*plu4187 *(> 1000 bp)	10.1-fold (± 1.8)	1	no	**ttgatt**cgacgatatt**-ttttatatt ****(insert)**
					**tt*a*att**taaatggtt--**ttttatatt**** (*plu1645*)**
					**ttgatt**agccaaataat**ttttat*c*tt**** (*plu2652*)**
					**ttgatt**tctttgtgt--**t*a*ttatatt ****(*plu2790*)**

7	*fadL *(440 bp)	6.4-fold (± 3.3)	1	no	**tttact**aaacaaaactgcaccgtc--**tttttttct**** (insert)**
					**tttact**aagcgcgttgtttactcatt***g*ttttttct**** (*spr*)**
					**tttact**ggatgatacacggtgctt--**tttttt*g*ct ****(*plu4229*)**

8	*tdh *(> 900 bp)	10.3-fold (± 3.9)	2	no	**tcgcca**gacacccgatcaccaa-**ttttaaaac ****(insert)**
					**tcgaca**taatcagctaacaatta**tttt*g*aaac**** (*mlc*)**
					**tc*t*cca**tttcattggaagggaag***c*tttaaaac**** (*tccC1)***

9	*plu0846/ppsA (plu2628) *(> 1000 bp)	13.6-fold (± 5.3)	1	no	**ttgcag**gtgcaggagcaggagaaaccgc**--ggtttaaat**** (insert)**
					**ttgca*t***gttgtaaaatattacaata**------ggttta*c*at**** (*plu1012*)**
					**ttgca*t***gttgtaaaatattacaata**------ggttta*c*at ****(*plu1017*)**
					**ttgc*c*g**tcgcgatgcatcttgaaatccatag**ggt*a*taaat**** (*ISPlu16A*)**

10	*plu4479/adhC *(> 1000 bp)	7.9 (± 3.4)	1	no	**ttcact**ctgcttaaggtatt**-----tgatgtact ****(insert)**
					**tt*t*act**ctcttgcgtccgactatcg**tgatgtac*a***** (*plu1579*)**

11	*plu1949-plu1950 *(944 bp)	10.2-fold (± 4.6)	1	yes	**tttatg**ccggttgcattaatt**cataaaaat ****(insert)**
					**tttatg**ccggttgcattaatt**cataaaaat**** (*plu1950*)**

12	*plu3608-plu3609 *(> 1000 bp)	4.5-fold (± 0.3)	1	yes	**ttgtta**aaaaatcgttatttttgg**ctatttaat**** (insert)**
					**ttgtta**aaaaatcgttatttttgg**ctatttaat**** (*plu3608*)**

13	*plu2635/plu0847 *(101 bp)	13.6-fold (± 5.3)	1	no	**ttgcag**gtgcaggagcaggagaaaccgc**ggtttaaat ****(insert)**

14	*agaR-agaZ *(961 bp)	3.3-fold (± 0.3)	1	yes	**ttgaat**ctcaaccct**atttagtct ****(insert)**
					**ttgaat**ctcaaccct**atttagtct**** (*agaZ*)**

15	*plu3688-plu3689 *(> 1000 bp)	4.0-fold (± 0.4)	1	yes	**ttatca**gaccatccggcagggcaag**gggaataaa ****(insert)**
					**ttatca**gaccatccggcagggcaag**gggaataaa ****(*plu3688*)**
					**ttatca**acgggctgttggtttaaa-**gg*a*aataaa**** (*plu2604*)**

16	*plu0643 *(855 bp)	3.7-fold (± 0.8)	1	no	**tggcct**gcccgaccaccgatt**ggatatatt ****(insert)**
					**t*t*gcct**tgtccctgtcaga--**ggat*g*tatt**** (*plu1165*)**

17	*glnD *(474 bp)	6.9-fold (± 2.1)	1	no	**tcgaac**accgttcacga**atatataat ****(insert)**
					**tc*c*aac**caaattgccg-**atat*t*taat**** (*eutA*)**

18	*walW *(127 bp)	3.2-fold (± 0.3)	1	no	**ttaatt**gaatcgttgagt**tgtgatact ****(insert)**
					**t*c*aatt**tggtaatacaac***a*gtgatact**** (*plu0801*)**
					**ttaa*a*t**atgccagtgtag**tctgatact**** (*plu2809*)**

The clones were identified by DFI and the sequence upstream the reporter gene was determined by DNA-sequencing. Fluorescence induction factors were determined by three independent measurements, experimental standard deviation is given in the brackets. The promoter motifs were identified with the Softberry BProm analysis tool and homologous promoter motifs were searched within the intergenic regions of the *P. luminescens *genome. The -10 and the -35 regions are highlighted in bold, and the mismatches compared to the identified promoter motifs are highlighted in italics. The genes downstream of the promoter motifs are marked.

In 17 of the 18 DNA-sequences, at least one putative promoter motif was identified upstream of the *mcherry *reporter gene (Tab. 1). Subsequently, we searched for similar promoter sequences in the intergenic regions of the *P. luminescens *genome, with one mismatch at the -10 or -35 regions considered and minimal deviation in spacers length preferred. Only those sequences directly upstream of coding genes were considered as putative promoters, which are presented along with downstream genes in Tab. 1. In eight cases, the putative promoter motif within the DNA-fragment exhibited similarities to more than one promoter in the *P. luminescens *genome (clone 1, clone 2, clone 6, clone 7, clone 8, clone 9, clone 15, and clone 18), whereas in one case (clone 13) no similar promoter motif could be predicted. In the remaining cases, a promoter motif was identified within the DNA-fragment that exhibited similarities to a *P. luminescens *promoter. Remarkably, the *plu3608 *promoter was identified experimentally (clone 12), and predicted computationally, by BProm within the sequence of clone 1. In summary, 29 promoters of different genes or operons were identified both directly by experimental result, and indirectly by BProm analysis (Tab. 1).

### Induction of predicted promoters *in vitro *and *in vivo*

The 29 genes or operons predicted to show induced expression in response to insect homogenate are summarized in Tab. 2. They comprise four genes encoding putative toxins (*tccC1*, *plu1645*, *plu2400*, and *plu4122*), six genes or operons possibly involved in metabolism (*agaZSVCD/plu0838/gatY*, *eutABC, hutUH, sucABCD*, *plu1864*, and *plu2604*), one regulator (*mlc*), three genes encoding DNA-modifying enzymes (*isplu16A*, *plu1165*, and *plu3688*), three genes encoding enzymes possibly involved in cell structural element synthesis (*spr*, *plu1463*, and *plu2790*), two genes encoding transporters (*plu1579*, *plu4229*) putatively involved in antibiotic resistance, one operon encoding an enzymes potentially involved in antibiotic synthesis *(plu3567-3561*), and nine genes or operons encoding proteins of unknown function (*plu0801*, *plu1012-1010*, *plu1017*, *plu1950*, *plu1672*, *plu2652*, *plu2809*, *plu3479*, and *plu3608*). To test induction of the predicted promoters, we cloned the 250–400 bp upstream sequence of the respective gene/operon directly upstream of the reporter. Furthermore, we cloned the upstream sequences containing the promoters of eight genes (*tcaA/B*, *tcdA1*, *tcaZ*, *tccA1*, *prtA*, *tcaZ*, *mcf1*, and *mcf2*) known to be involved in insect pathogenicity [18,21,41,42] upstream of the reporter as positive controls. *P. luminescens *carrying each one of these respective plasmids was cultivated in complex medium and complex medium containing *G. mellonella *larvae suspension, and the induction factor of each promoter was determined *in vitro *as described above. As shown in Tab. 2, most promoters exhibited confirmed induction. Only promoters for *plu1864 *and *plu3479 *showed no induction upon exposure to larvae homogenate. For those clones, when the presence of only one promoter was predicted, the induction factors of the predicted promoters were comparable to those of the original clones (e.g. clone 3: 2.8-fold induction comparable to *hutUH*: 2.5-fold induction, or clone 5: 4.3-fold comparable to *plu1463*: 6.5-fold), whereas for those clones with a prediction of more than one promoter the induction factors for each single promoter was more diverse. The highest induction factors observed were about 10-fold (*tccC1, plu1645, plu1012-1010*), whereas the lowest induction observed was 1.5-fold (*sucABCD*). Induction factors observed for the control promoters varied between 0.9 and 6.5, but it was only in the cases of *mcf *(6.0-fold) and *tcdA1 *(6.5-fold) that a clear induction was detectable under *in vitro *conditions, and a slight induction was observed in the cases of *tccA1 *(1.6-fold) and *prtA *(2.1-fold) (Tab. 2).

**Table 2:** Fluorescence induction of *P. luminescens *carrying different promoter-reporter gene fusions in presence of insect larvae homogenate

**Promoter of gene/operon**	**Putative function of the gene product(s)**	**Fluorescence Induction**
***Toxins***		
*tccC1 *(*plu4167*)	insecticidal toxin complex TccC1	**10.2-fold (± 2.0)**
*plu1645*	similar to photopexin A und B (hemopexin-domain)	**10.1-fold (± 4.5)**
*plu2400*	homologous to C-terminal region of dermonecrotic toxin (DNT) of *Pasteurella multocida*	**5.5-fold (± 1.9)**
*plu4122*	contains Fascin domain, function unknown	**6.3-fold (± 2.8)**

***Metabolism***		
*agaZSVCD/plu0839/gatY*		**3,3-fold (± 0.3)**
*agaZ *(*plu0833*)	tagatose-6-phosphate-Kinase	
*agaS (plu0834)*	tagatose-6-phosphate ketose/aldose isomerase	
*agaV *(*plu0835*)	PTS-system, N-acetylgalactosamine-specific IIB component 2 (EIIB-AGA')	
*agaC *(*plu0836*)	PTS-system, N-acetylgalactosamine-specific IIC component 1 (EIIC-AGA)	
*agaD *(*plu0837*)	PTS-System, N-acetylgalactosamine-specific IID component (EIID-AGA)	
*plu0838*	unknown, putative PTS permease	
*gatY *(*plu0839*)	tagatose-bisphosphate aldolase GatY	
*eutABC*		**3.5-fold (± 1.0)**
*eutA *(*plu2969*)	ethanolamine degradation	
*eutB *(*plu2970*)	ethanolamine-ammonia-lyase heavy chain	
*eutC *(*plu2971*)	ethanolamine-ammonia-lyase light chain	
*hutUH*		**2.5-fold (± 1.1)**
*hutU *(*plu3193*)	urocanate hydratase (urocanase)	
*hutH *(*plu3192*)	histidine ammonia lyase (histidase)	
*sucABCD*		**1.5-fold (± 0.4)**
*sucA *(*plu1430*)	α-oxoglutarate dehydrogenase E1 component	
*sucB *(*plu1431*)	dihydrolipoamid succinyltransferase component of α-oxoglutarate dehydrogenase complex (E2)	
*sucC *(*plu1432*)	succinyl-CoA synthetase β chain	
*sucD *(*plu1433*)	succinyl-CoA synthetase α-chain	
*plu1864*	similarities to phosphoenolpyruvat phosphomutase	1.0-fold (± 0.3)
*plu2604*	similar to glutarredoxin Protein YdhD	**5.0-fold (± 1.9)**

***Regulation***		
*mlc *(*plu2226*)	„making large colonies" protein, involved in regulation of sugar metabolism	**4.8-fold (± 1.7)**

***DNA-modification***		
*isplu16A *(*plu3160*)	transposase, IS630 family	**5.1-fold (± 1.0)**
*plu1165*	putative relaxase	**5.5-fold (± 2.0)**
*plu3688*	putative integrase/recombinase	**7.5-fold (± 3.1)**

***Synthesis of cell structure***		
*spr (plu2864)*	lipoprotein spr precursor	**3.0-fold (± 1.0)**
*plu2790*	similar to N-acetylmuramoyl-L-alanine amidase YbjR precursor of *Escherichia coli*	**4.7-fold (± 1.3)**
*plu1463*	similar to DNA inversion product and tail-fiber protein of lambdoid prophage	**6.5-fold (± 1.9)**

***Transport, antibiotic resistance***		
*plu4229*	multidrug-resistance permease	**2.4-fold (± 0.7)**
*plu1579*	similar to bacitracin permease of *E. coli*	**4.4-fold (± 1.4)**

***Antibiotic synthesis***		
*plu3567-3561*		**3.8-fold (± 1.2)**
*plu3567*	similar to N-formimidoyl fortimicin A synthase	
*plu3566*	similar to putative methylase and protoporphyrinogen oxidase	
*plu3565*	similar to class II aminotransferase and 5-aminolevulinic acid synthase	
*plu3564*	weakly similar to PapB protein and to chorismate mutase/prephenate dehydrogenase	
*plu3563*	similar to p-aminobenzoic acid synthase	
*plu3562*	similar to dehydrogenase PapC of *Streptomyces pristinaespiralis*	
*plu3561*	probable transport protein	

***Unknown function***		
*plu0801*	PrK012399-domain, similar to Plu1012 and Plu1017	**2.0-fold (± 0.3)**
*plu1012-1010*		**10.6-fold (± 4.2)**
*plu1012*	PrK012399-domain, similar to Plu0801 and Plu1017	
*plu1011*	„TIM-br_sig_trns"-Domain, putative sigma54-dependent transcriptional activator	
*plu1010*	unknown	
*plu1017*	PrK012399-domain, similar to Plu0801 and Plu1012	**6.6-fold (± 4.6)**
*plu1672*	similar to Plu1691 of *Photorhabdus*, function unknown	**2.0-fold (± 0.8)**
*plu1950*	similar to Plu2538 of *Photorhabdus*, function unknown	**3.0-fold (± 0.9)**
*plu2652*	function unknown	**4.0-fold (± 1.5)**
*plu2809*	similar to protein YcfD of *E. coli*, contains JmjC domain (pot. metalloenzym)	**3.0-fold (± 1.1)**
*plu3479*	unknown	1.1-fold (± 0.2)
*plu3608*	similar to protein Plu3387 of *Photorhabdus*, function unknown	**4.5 (± 0.3)**

***Controls***		
*mcf (plu4142)*	"makes caterpillars floppy" toxin	**6.0 (± 2.3)**
*mcf2 (plu3128)*	"makes caterpillars floppy" toxin 2	0.9 (± 0.2)
*tcdA1 (plu0962)*	insecticidal toxin complex TcdA1	**6.5 (± 1.0)**
*tcaA/B (plu0516)*	insecticidal toxin complex TcaA/TcaB	1.25 (± 0.2)
*tcaZ (plu0514)*	insecticidal toxin complex TcaZ	1.3 (± 0.3)
*tccA1 (plu4169)*	insecticidal toxin complex TccA1	1.6 (± 0.2)
*prtA (plu0655)*	alkaline metalloprotease PrtA	**2.1-fold (± 0.7)**

*P. luminescens *carrying the indicated reporter gene plasmid was grown in complex medium and in complex medium containing insect homogenate (*in vitro*). Each single reporter strain was tested on differential fluorescence induction in presence of insect homogenate. In the left panel, the genes or operons located downstream of the promoters tested are listed, along with putative functions for the gene products (middle panel). At the right panel, the fluorescence induction factors in presence of *G. mellonella *insect homogenate are shown. Enhanced activity is emphasized by bold numbers. Experimental standard deviation of at least three independent measurements is presented (in brackets).

**Figure 3:**
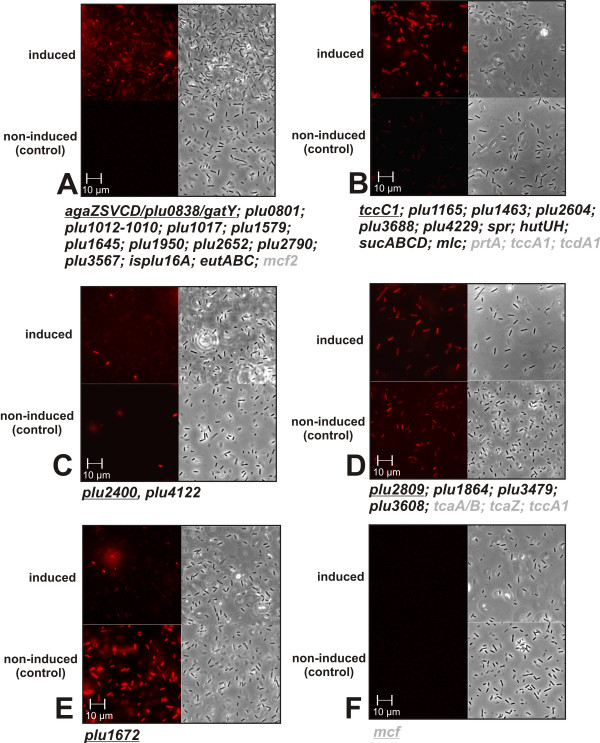
**Fluorescence induction of *P. luminescens *containing the different promoter-reporter gene fusions after growth in *G. mellonella***. *P. luminescens *TT01 transformants carrying plasmid pBR-Cherry with the promoters of the genes indicated were grown in complex medium, and *in vivo*, in *G. mellonella *larvae. Last instar larvae were infected with approximately 10,000 cells, and after 48–72 h the larvae were bled. The fluorescence of the bacteria present in the hemolymph was analyzed by microscopy, and compared to the fluorescence of cells incubated for an equal time in complex medium. Induced: cells grown in *G. mellonella*; non-induced: cells grown in complex medium. **A **- no fluorescence under non-induced conditions, fluorescence under induced conditions; **B **- fluorescence under non-induced conditions, but higher fluorescence under induced conditions; **C **- equal numbers of bright fluorescent cells under non-inducing and inducing conditions, but additional cells with low fluorescence intensity under inducing conditions;**D **- fluorescence was equal under non-inducing and inducing conditions; **E **- bright fluorescence under non-inducing conditions, and low fluorescence under inducing conditions; **F **- no fluorescence under non-inducing or inducing conditions. Strains carrying the indicated promoter-reporter gene fusions were grouped. The examples shown are underlined; the control promoters are shaded in grey. In the left panels, cells were observed through a fluorescence filter, in the right panels cells were observed with phase contrast.

To test the induction of the predicted promoters *in vivo*, *G. mellonella *was infected with ~10.000 *P. luminescens *cells harbouring the respective reporter plasmid. After 48–72 h the larvae were bled and the hemolymph was analyzed for the presence of fluorescing *P. luminescens *cells and compared with cells grown in complex medium (Fig. 3). For twelve promoters (*agaZSVCD/plu0838/gatY, plu0801, plu1012-1010, plu1017*,*plu1579*, *plu1645*,*plu1950, plu2652*, *plu2790*, *plu3567*,*eutABC*, and *isplu16A*) and one control (*mcf2*), no fluorescent cells were observed in complex medium, but bright fluorescent cells were clearly visible after passaging through *G. mellonella*, suggesting specific induction of the respective promoters within the insect host (Fig. 3A). In case of ten promoters (*tccC1*, *plu1165, plu1463*, *plu2604*, *plu3688, plu4229*, *spr*, *hutUH sucABCD*, and *mlc*) and three control promoters (*prtA*, *tccA1*, and *tcdA1*), fluorescent cells were visible when cells were grown in complex medium, but fluorescence was increased when cells were grown in *G. mellonella *larvae indicating specific up-regulation of the corresponding genes or operons in *P. luminescens *within the insect host (Fig. 3B). *prtA *transcription and PrtA protein production is growth phase dependent if cells were cultivated *in vitro *or in *G. mellonella *larvae [41]. This indicates that the fluorescence intensity induced by the *prtA *promoter can be correlated to transcription and translation of this gene, which can also be transferred to the other genes or operons subjected to promoter analysis here.

For promoters of *plu2400 *and *plu4122*, a few bright fluorescent cells were visible when cells were grown in complex medium; similar to what was observed for cells grown in *G. mellonella*. However, a large number of slightly fluorescent cells were observed *in vivo*, probably leading to the induction factors observed *in vitro *(Fig. 3C). For the promoters of *plu2809, plu1864*, *plu3479*, and *plu3608*, and the controls (*tcaA/B*, *tcaZ*, and *tccA1*) the number of fluorescent cells was similar in complex medium compared to growth in *G. mellonella *(Fig. 3D). This is in agreement with the induction factors determined *in vitro *for all promoters, except *plu2809 *and *plu3608*, for which a 3.0-fold or 4.5-fold respective induction was observed *in vitro *and no induction *in vivo*. For promoter *plu1672*, greater fluorescence was observed for the cells grown in complex medium compared to those grown in *G. mellonella *(Fig. 3E), which is in contrast to the *in vitro *determined induction factors. Furthermore, for the control promoter *mcf *no fluorescent cells were observed when cells were grown in either complex medium or in *G. mellonella *larvae, which was not in accordance with the 6.0-fold fluorescence induction observed *in vitro *(Fig. 3F). A possible explanation is that the growth curves were not completely identical for *in vitro *and *in vivo *conditions, leading to differences in fluorescence induction. Moreover, cells grown in *G. mellonella *are exposed to several different stress conditions compared to cells grown *in vitro*, which may affect the induction implied by *in vitro *data. Nevertheless, out of the 29 identified promoter motifs, promoters fluorescence induction was verified *in vitro *for 27, and verified *in vivo *in *G. mellonella *for 24. This indicated that a diluted insect homogenate is useful for the accurate screening of genes or operons that are induced by chemical signals produced in the insect host. Furthermore, the reliability of the bioinformatics analysis was determined to be between 80–90%.

Overall, different genes or operons of *P. luminescens *were identified to be up-regulated within the insect host. This includes increased expression of several toxin genes, including *tccC1 *and *plu2400*, which encode known and putative toxins (Tab. 2, Fig. 3). Another important feature of insect infection by *P. luminescens *appears to be a complex metabolic rearrangement. We identified a large number of metabolic genes or operons that were up-regulated in *P. luminescens *after insect infection. This includes operons that encode enzymes that are necessary for the degradation of tagatose, ethanolamine, or histidine, which were *agaZSVCD*, *eutABC*, and *hutUH*, respectively (Tab. 2, Fig. 3). Presumably, *P. luminescens *uses nutrients that are enriched in the insect host. Furthermore, the production of self-defence antibiotics and the development of resistance to other antibiotics appear to play an important role in the infection process as suggested by the up-regulation of certain genes upon insect infection that may be relevant to such processes (*plu3567-3561*, *plu4229*, and *plu1579*) (Tab. 2, Fig. 3). Moreover, the expression of genes encoding DNA-modifying enzymes is induced upon insect infection (Tab. 2), revealing enhanced rearrangements of the DNA and/or the genome under these conditions.

## Discussion

The most important findings of this study were the discovery of 18 different sequences present in the *P. luminescens *genome that are utilized for inducing gene expression upon infection of a host insect. We provide *in vitro*, *in silico *and *in vivo *evidences, including the evaluation of promoter utilization in a host infection model system, supporting our assertions pertaining to shifts in *P. luminescens *gene expression that accompany infection. Our results provide detailed insights into the changes that occur in bacteria in response to life cycle changes, which promote infection and pathogenicity.

We constructed a promoter-trap library of the entomopathogenic enterobacterium *P. luminescens *based on the Red Fluorescent Protein mCherry, and identified promoters that are utilized to stimulate gene expression in the presence of insect homogenate and during growth in *G. mellonella *larvae. Only four sequences represented original promoter motifs. In 13 cases, the sequences that promoted gene expression in response to the insect host environment were from known or predicted protein coding regions. A promoter pattern search for the identification of σ^70^-dependent promoter motifs was performed. In general, the σ^70 ^family of sigma factors comprises s-factors which are responsible for regulating a wide range of functions, all involved in sensing and reacting to conditions in the membrane, periplasm, or extracellular environment [40,43]. Using this bioinformatics approach we identified promoter motifs within 17 of the 18 DNA-sequences with similarities to 29 promoter motifs present in intergenic regions. For 27 promoters enhanced activity in the presence of insect homogenate was verified, and 24 of these promoters were validated *in vivo *for library expressing clones grown in *G. mellonella *larvae. This clearly showed that a DNA-fragment from a protein coding region with similarities to a promoter motif can act as a promoter when it is directly positioned upstream a reporter gene. We identified a total of 27 genes or operons that are up-regulated in *P. luminescens *when it enters the insect host, including those encoding: putative toxins, transport proteins, metabolic enzymes, synthases of structural elements, regulators and gene products of unknown function.

Four genes encoding putative toxins were identified to be induced upon insect infection: *tccC1*, *plu1645*, *plu2400*, and *plu4122. tccC1 *encodes the TccC subunit of an insecticidal toxin complex (Tc). The combination of three genes, *tcdA*, *tcdB*, and *tccC*, is essential for oral toxicity to *M. sexta *when expressed in *E. coli*. TcdAB is believed to be the major insecticidal complex, whereas TccC appears to be a stand alone toxin [44] or an enhancer toxin for TcdAB [45]. The genome of *P. luminescnes *TT01 contains seven *tccC *homologues (*tccC1-tccC7*), and it is hypothesized that different homologues might be required for infection of different insect species so that overkill may be achieved. *E. coli *cells expressing the *tccC1 *homologue of *X. nematophila *became highly virulent towards *G. mellonella*, underlining the hypothesis that TccC1 toxin complex might play a major role in virulence [46]. Furthermore, *tccC1 *is located downstream of two other *tc *genes, *tccB1 *and *tccA1; *the promoter of the latter one was tested as a control and exhibited enhanced activity in *G. mellonella*.

The protein Plu1645 consists of N-terminal HX and C-terminal PA-IIL domains. PA-IIL-proteins bind Fucose-Lectin II, which is important for virulence of *P. aeruginosa *[47]. Furthermore, Plu1645 shares similarities to PpxA and PpxB of *P. luminescens*, which encode photopexin A and B, two proteins that consist of two HX-domains connected to a PA-IIL domain. The photopexins in *P. luminescens *represent the first hemopexin-like proteins found in prokaryotes and are believed to be involved in iron scavenging [48], and therefore in virulence as well.

Plu2400 represents a novel *Photorhabdus *toxin candidate involved in insect infection. The protein does not contain any conserved protein domain, but it shares similarities with the C-terminal region of the dermonecrotic toxin of *Pasteurella multocida *(PMT). PMT is a cytotoxin that stimulates mitogenesis and cytoskeletal reorganization [49,50], and modulates host cell cycle progression [51]. Referring to the size differences of Plu2400 (568 aa) and PMT (1285 aa), and the limited homology of Plu2400 in the C-terminal region of PMT, it can be assumed that the mode of action of Plu2400 might differ from PMT.

Another putative novel *Photorhabdus *toxin is Plu4122, which contains a Fascin-domain. Fascins are eukaryotic proteins that bundle actin filaments, thereby affecting cell division, adhesion and motility. Eukaryotic fascins consist of four connected Fascin-domains, and are highly regulated at the transcriptional, translational and posttranslational levels by Protein Kinase C (PKC) (see [52,53] for review). Plu4122 contains only one Fascin-domain containing a putative PKC phosphorylation site, suggesting that it could deregulate eukaryotic actin bundling by attenuating fascin phosphorylation, resulting in disorganization of the actin cytoskeleton.

The number of metabolic genes or operons identified to be induced in *P. luminescens *upon insect infection suggests alterations in metabolism upon host infection. Histidine and phosphatidylethanolamine were discussed before to be potential metabolites used by *P. luminescens *as substrates for growth within the insect host [24], which is consistent with the finding that the *hutUH *and *eutABC *operons encoding the respective degradation enzymes are induced upon insect infection. Furthermore, histidine is one of the most abundant free amino acids in the *Hyalophora gloveri *fat body [54]. The Mlc protein is involved in phosphotransferase-system (PTS) driven sugar uptake by acting as a repressor for several PTS genes encoding the components for the uptake of specific sugars as substrates [55]. The up-regulation of *mlc *in *P. luminescens *within the insect host further emphasizes that metabolism is significantly changed upon infection. Furthermore, we found that the promoter of the *suc*-operon encoding α-oxoglutarate synthase and succinyl-CoA synthase is slightly induced in *P. luminescens *within the insect host. The specific up-regulation of the tricarboxylic acid cycle (TCA) enzymes within a host has been described also for other microorganisms. For example, the *suc*-operon is induced in *V. cholerae *during host infection [56,57], and a complete TCA cycle is required for *S. typhimurium *virulence [58]. Since genes of different TCA cycle enzymes were observed as induced in several pathogens, including *Listeria monocytogenes*, *Shigella flexneri*, and *Yersinia enterocolitica *upon host infection, it is hypothesized that the up-regulation of the TCA cycle and the correlating induced profit of energy is important for growth of bacteria within hosts, which was predicted for *P. luminescens *before [34,24].

Antibiotic production by *P. luminescens *plays a global role in the insect host, possibly to a time point when other bacteria, e.g. from the insect gut, are released into the cadaver and may compete with *P. luminescens *for nutrients. The total number of microbes in the hindgut of soil invertebrates can reach a titre of 10^11^/ml [59], and these are all possible competitors of *P. luminescens*. The *plu3567-3561 *operon encodes enzymes that are homologues of those from known antibiotic biosyntheses pathways [60,61], and it is induced upon entering the insect host. It is proposed that *plu3567-3561 *encodes enzymes for the biosynthesis of a thus far unknown antibiotic that is used as a self-defence mechanism by *P. luminescens *for selecting against competing microbes during the infection of the insect host.

Both the synthesis of and the resistance to antibiotics appears to be increased by *P. luminescens *in the insect host. Bacteria produce Multiple-Drug-Resistance (MDR) efflux pumps that protect against antibiotics and other substances, such as dyes and detergents [62]. Recently, it has been reported that MDR permeases also export host-derived antimicrobial agents, and it has been suggested that the physiological role of these systems is to evade naturally produced antimicrobial molecules, thereby allowing the bacterium to survive in a special ecological niche or host [63]. With the up-regulation of the *plu4229 *gene, putatively encoding an MDR permease, *P. luminescens *might not only defend against antibiotics produced by bacterial competitors, but also against antimicrobial agents produced by the host *G. mellonella*.

Rearrangements of the cell structure might be important for *P. luminescens *survival within *G. mellonella*. Three genes (*spr*, *plu2790*, and *plu1463*) encoding enzymes possibly involved in the synthesis of cell structure components were found to be up-regulated in *P. luminescens *in the insect host. Plu2790 is similar to the periplasmic lipoprotein YbjR (AmiD) of *E. coli*, which cleaves anhydromuropeptide anhMurNAc-L-Ala [64]. Uncleaved anhydromuropeptides released into the medium trigger many types of bacterial interactions, including symbiosis and interactions between microorganisms, as well as the induction of host innate immune responses [65,66]. AmiD is used to avoid innate immune responses in these environments by degrading these compounds in the periplasm [64]. *P. luminescens *might therefore use the AmiD homologue Plu2790 for the degradation of anhydromuropepdides as a strategy avoiding or silencing the insect's immune response after infection. The *spr *gene (*plu2864*), which is also up-regulated upon insect infection, represents a precursor in lipoprotein biosynthesis, which correlates with the increased production of Plu2790. In general, bacterial lipoproteins are involved in a wide variety of cellular functions, such as formation and stabilization of the cell surface structure, substrate transport, antibiotic resistance and cell signaling [67].

*plu1463 *encodes a tail-fibre protein of a lambdoid prophage that is induced in *P. luminescens *upon entering the insect. Prophage elements encode proteins with domains of various important functions, including toxicity, virulence, bacteriophage resistance, DNA-modification and antibiotic resistance [68]. The function of *plu1463 *in *P. luminescens *during insect infection is still unclear. Generally, phage leave there hosts when cells are under stress [69]. A possible explanation might be that the phage-related genes are expressed when the phage enters the lytic cycle, which in turn happens when *P. luminescens *enters the insect host. The induction of another gene, *plu2606 *encoding a glutarredoxin-like protein might be involved in persistence of stress within the hemolymph where cells are massively exposed to oxidative stress, which could also lead to stimulating phage transition out of dormancy.

When cells are exposed to different stresses, one consequence is DNA damage [70]. The hypothesis that *P. luminescens *is exposed to stress within the insect is supported by the enhanced expression of *plu3160*, which encodes a transposase, and *plu3688*, which encodes a putative integrase/recombinase. Furthermore, expression of *plu1165 *is induced under this condition. The corresponding protein, Plu1165, contains N-terminal TraI_2 and C-terminal DUF1528 domains, which is a domain-combination that is similar to putative relaxases [71]. Relaxases nick duplex DNA in a site- and strand-specific manner by catalyzing a transesterification reaction, and are commonly involved in conjugation processes [72]. Upon insect infection, *P. luminescens *undergoes a phenotypic switch from the primary to the secondary phase variant [4], but a reorganization of the genome of *P. luminescens *was never observed at that point [73,74]. We identified genes encoding DNA-modifying enzymes as up-regulated upon insect infection, which supports the hypothesis that *P. luminescens *undergoes multiple processes of DNA rearrangement upon phase variant switching within the insect host.

Genes or operons encoding products of unknown function have been identified as up-regulated in *P. luminescens *while in the insect host, and these are promising pathogenicity candidate factors. Remarkably, among these were: *plu0801*, *plu1012*, and *plu1017*, which encode proteins harbouring a PrK012399-domain, and are the only three proteins in *P. luminescens *with this domain.

## Conclusion

A DFI approach using a reporter gene library of a bacterial symbiont or pathogen with host homogenate as inducer is useful to screen for genes or operons that are up-regulated within this host, demonstrated here by the use of a *P. luminescens *promoter-trap library and wax moth homogenate as inducer. We show that not only native promoters but also intragenic DNA-fragments comprising promoter-similar motifs induce reporter gene expression. Genes or operons that are potentially up-regulated can be identified by comparison of the promoter-similar motifs to promoters in the genome using bioinformatics tools, whereas enhanced activity for those predicted promoters has to be verified. In our study, enhanced activity was confirmed *in vitro *for 93% and *in vivo *for 83% of those predicted promoters. Since the screen was performed with insect homogenate, the signals that regulate the expression of the genes or operons identified by this DFI approach are chemical signals present in the insect body. Genes or operons encoding proteins that are part of metabolic pathways, like *hutUH *or *eutABC*, are most likely up-regulated in response to substrates available the insect body. Moreover, *P. luminescens *has a variety of LuxR-like receptors that are predicted to recognize signaling molecules, such as hormones, produced by the host [24]. Two of the LuxR-like receptors are similar to LuxR of *Vibrio fischeri*, but no LuxI was found excluding the self-production of homoserine lactones [24]. Bacteria and their hosts communicate with each other through an array of hormones and homoserine lactones, indicating that quorum-sensing signaling is not restricted to bacterial cell-to-cell communication [33]. Hormones produced by the insect or homoserine lactones produced by the insect gut flora could be signals used by *P. luminescens *for adapting gene expression to pathogenesis.

## Methods

### Bacterial strains and growth conditions

The *P. luminescens *strain used was TT01 [75].*E. coli *strains JM109 [*recA1 endA1 gyrA96 thi hsdR17 supE44 relA1 Δ (lac-proAB)/*F'*traD36 proA*^+^*B*^+^*lacI*^*q*^*lacZ*ΔM15] and JM110 [*rpsL *(Str^R^) *thr leu thi-1 lacY galK galT ara tonA tsx dam dcm supE44 *Δ (*lac-proAB*)/F'*traD36 proA*^+^*B*^+^*lacI*^*q*^*lacZ*ΔM15] were used [76] as carriers for the plasmids described and for cloning. *E. coli *strains were grown aerobically at 37°C in LB medium [10% (w/v) peptone, 5% (w/v) yeast extract, 10% (w/v) NaCl], whereas *P. luminescens *was grown aerobically at 30°C in CASO medium [10% (w/v) peptone of casein, 5% (w/v) peptone of soy flour, 5% (w/v) NaCl]. For preparation of solid media, 1.5% (w/v) agar was added. Within the screen, *P. luminescens *was cultivated aerobically in Schneider's insect cell medium [77]. Ampicillin or carbenicillin was added to a final concentration of 100 μg/l. Host-inducing conditions were simulated by preparation of a *G. mellonella *larvae suspension in Schneider's medium. For this purpose, one animal (45–55 mg) was surfaced-sterilized by bathing in 70% (v/v) ethanol followed by sterile water, and killed by removing the head. The body was cut into pieces with a sterile scalpel, and the pulp was diluted in 50 ml of Schneider's medium followed by continuous mixing for 15 min on a vortex mixer. Tissue debris was removed by centrifugation at 5000 rpm (4°C) for 20 min.

### Plasmid construction

For construction of a promoter-less *mcherry *reporter gene plasmid, *mcherry *was amplified using plasmid p2641 (M. Engstler, TU Darmstadt, Germany) as a template with primers that added a *Msc*I/*Bam*HI/*Xma*I restriction site to the 5' end, and a *Hin*dIII restriction site to the 3' end of the PCR product. The DNA-fragment was cut with restriction endonucleases *Hin*dIII and *Msc*I, and ligated into equally treated vector pBR322, resulting in plasmid pBR-Cherry. For construction of plasmid pBR-Cherry-rpsM, the promoter region of *rpsM *was amplified with primers adding a 5' *Msc*I and a 3' *Xma*I site to the DNA-fragment using genomic *P. luminescens *DNA as template. The PCR product was cut with *Msc*I and *Xma*I, and ligated into equally treated vector pBR-cherry, resulting in the plasmid pBR-Cherry-rpsM product.

For construction of the *P. luminescens *promoter-trap library using *mcherry *as a reporter gene, genomic DNA of *P. luminescens *was cut with *Alu*I and *Dra*I, respectively, resulting in a predicted population of fragments that were 300–700 bp in size. The fragments were mixed and ligated randomly into *Sma*I linearized vector pBR-cherry, such that the DNA-fragments were directly cloned in front of the *mcherry *reporter gene. Cloning steps were performed using *E. coli *JM109, and the library was later brought into *P. luminescens *by electroporation.

The promoters of the identified genes in the screen were cloned directly upstream of the *mcherry *reporter gene. For this purpose, DNA-fragments comprising 250–400 bp upstream of the respective gene were amplified by PCR with primers adding a 5' *Msc*I and a 3' *Xma*I, or a 5' *Bam*HI site and a 3' *Xma*I site to the amplified fragment using genomic DNA of *P. luminescens *as template. The DNA-fragments were cut with the appropriate restriction enzymes and ligated into equally treated vector pBR-Cherry.

Verification of all plasmids was performed by restriction analysis and by DNA-sequencing.

### Competent cells and transformations

*E. coli *cells were made chemically competent and transformed as described elsewhere [78]. *P. luminescens *was made electrocompetent and transformed by electroporation. Cells of *P. luminescens *were cultivated aerobically in CASO medium at 30°C up to an OD_600 _of 0.8–1.0. Then, cells were incubated on ice before they were harvested by centrifugation at 4°C. The cell pellet was resuspended in the same volume of ice-cold 10% (v/v) glycerol and collected again by centrifugation. Cells were then washed in 1/2 starting volume, and then in 1/20 starting volume of 10% (v/v) glycerol, and then resuspended in 1/300 starting volume of 10% (v/v) glycerol. For the following electroporation step, 60 μl of cell suspension were mixed with 100 ng plasmid-DNA (pBR-Cherry derivatives) or 3–7 μg plasmid-DNA (pBR-Cherry-PluLib), incubated on ice for 1 min, and then transferred into 0.2 cm electroporation cuvettes. Electroporation was performed with a pulse of 2500 V for 4–6 msec. Subsequently, cells were removed from the cuvettes by flushing with 1 ml CASO medium, and incubated aerobically at 30°C for 1 h. The complete transformation samples were plated on appropriate agar plates and incubated at 30°C for two days.

### Infection of insect larvae, re-isolation of bacteria and microscopy

*Galleria mellonella *larvae were incubated on ice for 10 min to reduce movements and surface sterilized in a 70% (v/v) ethanol bath followed by a bath of sterile water. Larvae were infected with *P. luminescens *cell suspensions by injection of 10 μl cell suspensions subcutaneously using a sterilized micro syringe (Hamilton 1702 RN, 25 μl), and incubated at 25°C for 2–3 days. For re-isolation of the bacteria, the dead animals were surface sterilized and the cuticula was cut with a sterile scalpel. The out coming hemolymph was harvested, pipetted into CASO medium and subsequently analyzed by fluorescence microscopy using a Leica DFC350 FXR2 microscope (Leica Microsystems, Wetzlar). Since the fluorescence of *P. luminescens *was low, pictures were recorded with an Andor iXon^+ ^high sensitivity camera (Andor Technology, Puchheim).

### Fluorescence screening

Incubation of the promoter-trap library clones for screening was carried out in 96-well microtiter plates (black plates with transparent bottom sites). Single clones of the *P. luminescens mcherry*-promoter-trap library were incubated aerobically at 30°C both under non-induced conditions in Schneider's medium and under induced conditions in Schneider's medium containing larvae suspension (100 μl). The measurement of OD_600 _and fluorescence was performed in an "Infinite 500" Plate reader (Tecan, Austria) with an excitation wavelength of 560 nm (20 nm bandwidth) and an emission wavelength of 610 nm (20 nm bandwidth). The integration time was set to 20 μs and the number of measurements was 10 for measurement of fluorescence and for optical density. Analysis and conversion of the raw data into induction factors was performed using the "Magellan" software (Tecan, Austria). Each plate contained four negative controls: two medium blanks (solely Schneider's medium and Schneider's medium containing larvae suspension reflecting non-induced and induced conditions), and two fluorescence blanks (*P. luminescens *pBR-cherry harbouring the promoter-less *mcherry *reporter), under non-induced and induced conditions. First, the raw data were corrected with the respective medium blank. Then, the respective fluorescence value was normalized with the optical density, and then corrected by the fluorescence blank. The correlation and linearity of fluorescence and optical density was verified before by testing different numbers of *P. luminescens *cells carrying plasmid pBR-Cherry-rpsM. The values of the normalized fluorescence under non-inducing and under inducing conditions were divided by the value of the non-inducing conditions, which gave induction factors in the wells under inducing conditions, and an induction factor of one in each of the wells under non-inducing conditions. Those clones with an induction factor of ≥ 2.5 under inducing conditions were selected for verification, and were re-inoculated three more times. Verified positive clones consistently showed an induction-factor of ≥ 2.5 in each replicate.

The plasmids of the respective clones were isolated, but the material prepared from *P. luminescens *was not useful for DNA-sequencing. Therefore, *E. coli *JM109 was transformed with each plasmid, which was then re-isolated. The sequence of the DNA-fragments upstream of *mcherry *was analyzed by DNA-sequencing using an antisense primer annealing within the *mcherry *cassette.

### Bioinformatics tools

For promoter sequence identification, the BLAST analysis tool on the PhotoList server of the Pasteur Institute [79] was used. For the identification of possible promoter motifs within entire gene sequences, the respective DNA-sequences were analyzed with the BProm tool, which is a bacterial σ^70 ^promoter recognition program with about 80% accuracy and specificity on the Softberry server [80]. Then, the respective DNA pattern within the putative -35 and the -10 sequence motifs, as well as their lengths, were searched against non-coding regions of the *P. luminescens *genome using the PATTERN SEARCH tool on the PhotoList server [79]. In the -10 and -35 regions, a maximum of one mismatch was allowed within the sequences present in non-coding regions of the genome. Patterns with a minimal deviation in the length of spacers were preferred (0–2 bp). Only when the identified sequence was upstream of a putative gene in the correct direction the identified sequence motif was marked as a putative promoter.

## Abbreviations

DFI: Differential Fluorescence Induction; Tc: Insecticidal Toxin Complex; MDR: Multiple Drug Resistance; JHE: Juvenile Hormone Esterase; PMT: *Pasteurella multocida *toxin; PKC Protein kinase C; PTS: Phosphotransferase-system; TCA: Tricarbolic acid cycle; AI-2: autoinductor-2.

## Authors' contributions

LS constructed the *P. luminescens *promoter-trap library and performed all preliminary tests for the screen application. AM performed the library screen, the bioinformatics analyses and the promoter cloning. KJ was involved in data interpretation and evaluation. RH coordinated the project, supervised and evaluated the experiments, performed the fluorescence microscopy, and drafted the manuscript. All authors have read and approved the final manuscript.
